# Changes in the cohort composition of turner syndrome and severe non-diagnosis of Klinefelter, 47,XXX and 47,XYY syndrome: a nationwide cohort study

**DOI:** 10.1186/s13023-018-0976-2

**Published:** 2019-01-14

**Authors:** Agnethe Berglund, Mette Hansen Viuff, Anne Skakkebæk, Simon Chang, Kirstine Stochholm, Claus Højbjerg Gravholt

**Affiliations:** 1 Department of Endocrinology and Internal Medicine, Aarhus University Hospital, Palle Juul-Jensens Boulevard 99, 8200 Aarhus N, Denmark; 2 Department of Molecular Medicine, Aarhus University Hospital, Brendstrupgaardsvej 21A, 8200 Aarhus N, Denmark; 3 Department of Clinical Genetics, Odense University Hospital, J.B. Winsløws Vej 4, 5000 Odense C, Denmark; 4 Unit for Thrombosis Research, Department of Regional Health Research, University of Southern Denmark, Odense, Denmark; 5 Department of Clinical Biochemistry, Hospital of South West Jutland, Finsensgade 35, 6700 Esbjerg, Denmark; 6 Department of Pediatrics, Center of Rare Diseases, Aarhus University Hospital, Palle Juul-Jensens Boulevard 99, 8200 Aarhus N, Denmark

**Keywords:** Turner syndrome, Klinefelter syndrome, Triple X syndrome, Double Y syndrome, Prevalence, Incidence, Age at diagnosis

## Abstract

**Background:**

Knowledge on the prevalence of sex chromosome abnormalities (SCAs) is limited, and delayed diagnosis or non-diagnosis of SCAs are a continuous concern. We aimed to investigate change over time in incidence, prevalence and age at diagnosis among Turner syndrome (TS), Klinefelter syndrome (KS), Triple X syndrome (Triple X) and Double Y syndrome (Double Y).

**Methods:**

This study is a nationwide cohort study in a public health care system. The Danish Cytogenetic Central Registry (DCCR) holds information on all karyotypes performed in Denmark since 1961. We identified all individuals in the DCCR with a relevant SCA during 1961–2014; TS: *n* = 1156; KS: *n* = 1235; Triple X: *n* = 197; and Double Y: *n* = 287. From Statistics Denmark, which holds an extensive collection of data on the Danish population, complete data concerning dates of death and migrations in and out of Denmark were retrieved for all individuals.

**Results:**

The prevalence among newborns was as follows: TS: 59 per 100,000 females; KS: 57 per 100,000 males; Triple X: 11 per 100,000 females; and Double Y: 18 per 100,000 males. Compared with the expected number among newborns, all TS, 38% of KS, 13% of Triple X, and 18% of Double Y did eventually receive a diagnosis. The incidence of TS with other karyotypes than 45,X (*P* < 0.0001), KS (*P* = 0.02), and Double Y (*P* = 0.03) increased during the study period whereas the incidence of 45,X TS decreased (*P* = 0.0006). The incidence of Triple X was stable (*P* = 0.22).

**Conclusions:**

The prevalence of TS is higher than previously identified, and the karyotypic composition of the TS population is changing. Non-diagnosis is extensive among KS, Triple X and Double Y, whereas all TS seem to become diagnosed. The diagnostic activity has increased among TS with other karyotypes than 45,X as well as among KS and Double Y.

## Introduction

Sex chromosome abnormalities (SCAs) - Turner syndrome (TS [45,X]), Klinefelter syndrome (KS [47,XXY]), Triple X syndrome (Triple X, [47,XXX]) and Double Y syndrome (Double Y,[47,XYY]) - are estimated to affect approximately 1 per 400 births [1]. A number of representative cytogenetic surveys were conducted years ago on newborns in various countries. Previously, we pooled data from these surveys in order to estimate the prevalence of TS, KS, Triple X and Double Y [2–5] which were; TS: 50 per 100,000 newborn females [24 TS among 48,744 newborn females] [6–10]; KS: 152 per 100,000 newborn males [84 KS among 55,212 newborn males] [6, 7, 10–13]; Triple X: 84 per 100,000 newborn females [62 Triple X among 73,990 newborn females] [4]; 4) Double Y: 98 per 100,000 newborn males [51 Double Y among 52,004 newborn males] [7, 10, 12, 14, 15]. Estimates are, however, subject to much uncertainty.

The presence of a SCA may affect individuals at multiple organ levels, but the range of affection is very wide. Abnormal and delayed puberty as well as infertility are, however, distinctive features in individuals affected by TS or KS [16, 17]. Previously, we and others, have reported an almost four-fold increased mortality [3–5, 18, 19] as well as increased morbidity associated to a wide range of diseases [18, 20] in individuals affected by SCAs compared to age and sex-matched controls. Further, we have reported a reduced socioeconomic status in individuals affected by a SCA with lower education (except for TS), increased risk of being retired from the labor market, lower income, and reduced likelihood of living in a relationship [21–24].

Delayed or even non-diagnosis of SCAs is common. Individuals not diagnosed in infancy often do not receive a diagnosis until years after relevant medical therapy should have been initiated to alleviate symptoms [4, 5, 25–27]. Further, delayed diagnosis prevents timely screening and intervention for common associated health problems as well as learning and behavioral disabilities [28, 29]. Thus, health related and social consequences of non-diagnosis and delayed diagnosis are a continuous concern.

In the present study we aimed to investigate change over time in incidence, prevalence, and age at diagnosis in a national cohorts of SCAs.

## Material and methods

### Registries and cases

The Danish Civil Registration System was established in 1968 and since then all persons residing in Denmark have been assigned a unique 10-digit civil personal registration (CPR) number. The last digit in the CPR number allows identification of the persons’ officially registered sex (odd = male; even = female). Further, the CPR number allows accurate matching of data from different data sources [30]. The Danish health care system is a public tax funded system ensuring all citizens free and equal health care access.

The Danish Cytogenetic Central Registry (DCCR) was established in 1968, and holds data on all pre-and postnatal karyotypes performed in Denmark since 1961. Only postnatally diagnosed individuals or prenatally diagnosed individuals, subsequently postnatally confirmed, are included in the present study. Data in the DCCR includes: 1) CPR number; 2) karyotype; 3) date of birth; and 4) date of karyotyping. The DCCR holds no information on phenotype or reasons for performing a karyotype. Information concerning degrees of mosaicism is not available.

The DCCR was searched for all cases diagnosed with a karyotype compatible with TS, KS, Triple X or Double Y during 1961–2014. The distribution of accepted karyotypes for each syndrome are presented in Table 1. We considered females with a 45,X mosaic composition to have a phenotype most consistent with TS and these cases were thus included in the TS group. Females with more than three X chromosomes (e.g. 48,XXXX) and males with more than two Y chromosomes (e.g. 48,XYYY) were included in the Triple X and Double Y group, respectively. Cases with an autosomal aneuploidy were included in their respective group of SCA. Data were retrieved from the DCCR in October 2015.

**Table 1:** Distribution of karyotypes among Turner syndrome, Klinefelter syndrome, Triple X and Double Y syndrome

Karyotypes	Number
Turner syndrome	1156
45,X	422
45,X/46,XX	287
Karyotypes containing an isochromosome: 45,X/46,I(X) and equivalents	117
Karyotypes containing Y chromosome material: 45,X/46,XY; and equivalents	47
Other	283
Klinefelter syndrome	1235
47,XXY	1080
47,XXY/46,XY	78
47,XXY/46,XX/46,XY; 46,XY/47,XXY/48,XXXY; and equivalents	32
Karyotypes with an autosomal aneuplodi: 48,XXY,+ 18; 48,XXY,+ 21	5
Other	40
Triple X syndrome	197
47,XXX	104
47,XXX/46,XX	58
48,XXXX; 49,XXXXX	10
Karyotypes with an autosomal aneuplodi: 48,XXX + 18; 48,XXX + 21; and quivalents	10
Other	15
Double Y syndrome	287
47,XYY	206
47,XYY/46,XY	26
Karyotypes containing an isochromosome: 46,XY/47,XY,+I(Yq); 45,X/47,XY,+I(Y)/46,XY; and equivalents	6
48,XXYY; 48,XYYY; and equivalents	35
Other	14

The Causes of Death Registry holds information on all deaths since 1970 including date of death and primary and auxiliary cause of death [31]. We retrieved data on all deceased cases during 1970 to December 31, 2014. Further, from the Civil Registration system complete data on migrations in and out of Denmark were retrieved.

Statistics Denmark is a state institution under the Ministry of Economic Affairs and the Interior and collects data on the Danish population. From Statistics Denmark (https://www.dst.dk/en), annually data concerning the number of females and males living in Denmark were retrieved. Likewise was annual data on the Danish birth cohorts.

### Statistics

We distinguish between population-based prevalence and prevalence among newborns. In the following, we solely use the term “prevalence” when describing the prevalence of SCAs among newborns. All cases were incident the year they were diagnosed and prevalent the year they were born.

The population-based prevalence was estimated as the annual number of cases being alive in Denmark each year during the study period (1970–2014). 1970 was chosen as start of the observation period to avoid confounding from a run-in phase of the DCCR. We defined cases diagnosed prior to 1970 to be prevalent in 1970. Deceased cases were excluded the first year following death. Emigrated cases were excluded the first year following emigration, if emigration was not followed by subsequent immigration. A population-based prevalence was also estimated using the expected prevalence of the individual SCAs. Linear extrapolation were used to estimate when the observed and the expected population-based prevalence equals each other.

Incidence was estimated as the average number of diagnosed cases per million females (TS and Triple X) or males (KS and Double Y) in the background population each year during the study period. Cases diagnosed prior to 1970 were not included in this analysis.

Prevalence was estimated as the average number of cases being born per 100,000 newborn females (TS and Triple X) or males (KS and Double Y) in the background population. Cases were clustered according to 5-year calendar time periods, and the number of diagnosed cases per 5 years was divided by the sum of their five respective birth cohorts. Data on the Danish birth cohorts are available since 1901, thus observation periods started in 1901, and cases born prior to 1901 (TS: *n* = 5; KS: *n* = 11; Triple X: *n* = 2; and Double Y: n = 1) were thus not included in this analysis. All observation periods ended in 2014.

Time trend in incidence was analyzed using Poisson regression. Time trend in age at diagnosis during the study period was analyzed using linear regression. Differences in age at diagnosis among subgroups within one syndrome or among different syndromes were analyzed using Kruskal-Wallis test. *P*-values < 0.05 was considered statistically significant. Analyzes were made in StataCorp 13.1 and 15.1 for Windows.

## Results

During 1961–2014 a total of 2875 individuals were diagnosed and recorded in the DCCR with a karyotype compatible with TS (*n* = 1156); KS (*n* = 1235); Triple X (*n* = 197); or Double Y (*n* = 287) (Table 1). Year of diagnosis did not differ among the syndromes (*P* = 0.07).

### Population-based prevalence

**Fig. 1:**
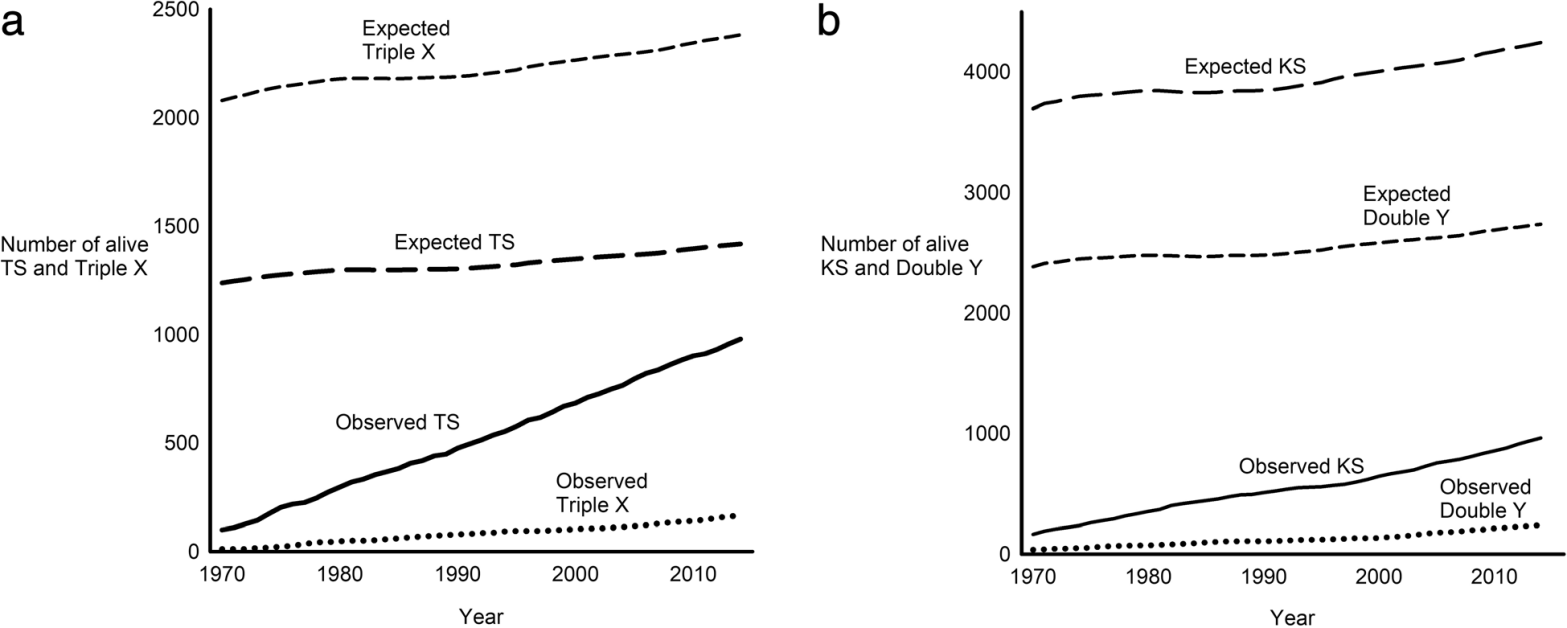
Absolute prevalence of Turner syndrome, Klinefelter syndrome, Triple X syndrome and Double Y syndrome in the Danish population. The observed number of **a** Turner syndrome (TS) and Triple X syndrome and **b** Klinefelter syndrome (KS) and Double Y syndrome (Double Y) being alive during 1970–2014 are illustrated by solid and dotted lines. Deceased or emigrated individuals were subtracted. Dashed lines indicate the expected number assuming a true prevalence of 1) 50 TS per 100,000 at birth; 2) 84 Triple X per 100,000 at birth; 3) 152 KS per 100,000 at birth; and 4) 98 Double Y per 100,000 at birth as well as a similar mortality as in the general population

The population-based prevalence of TS, KS, Triple X and Double Y increased during the study period (Fig. 1). Compared to the expected population-based prevalence, 70% of TS (980 out of 1418); 23% of KS (962 out of 4244); 7% of Triple X (165 out of 2381); and 9% of Double Y (239 out of 2736) were diagnosed and alive by the end of the study.

Linear extrapolation of the observed and expected population-based prevalence of TS show that these theoretically equals each other in 2039 – in other words, all live TS will be diagnosed in 2039, given the current diagnostic practices continue unaltered, given the expected population-based prevalence is correct, and given the mortality rate is identical in TS and in the background population. For KS, this point in time was estimated to the year of 2471. Theoretically, the expected and observed prevalence of Triple X and Double Y will continue to diverge owing to a greater increase in the expected prevalence than in the observed prevalence.

### Incidence

**Fig. 2:**
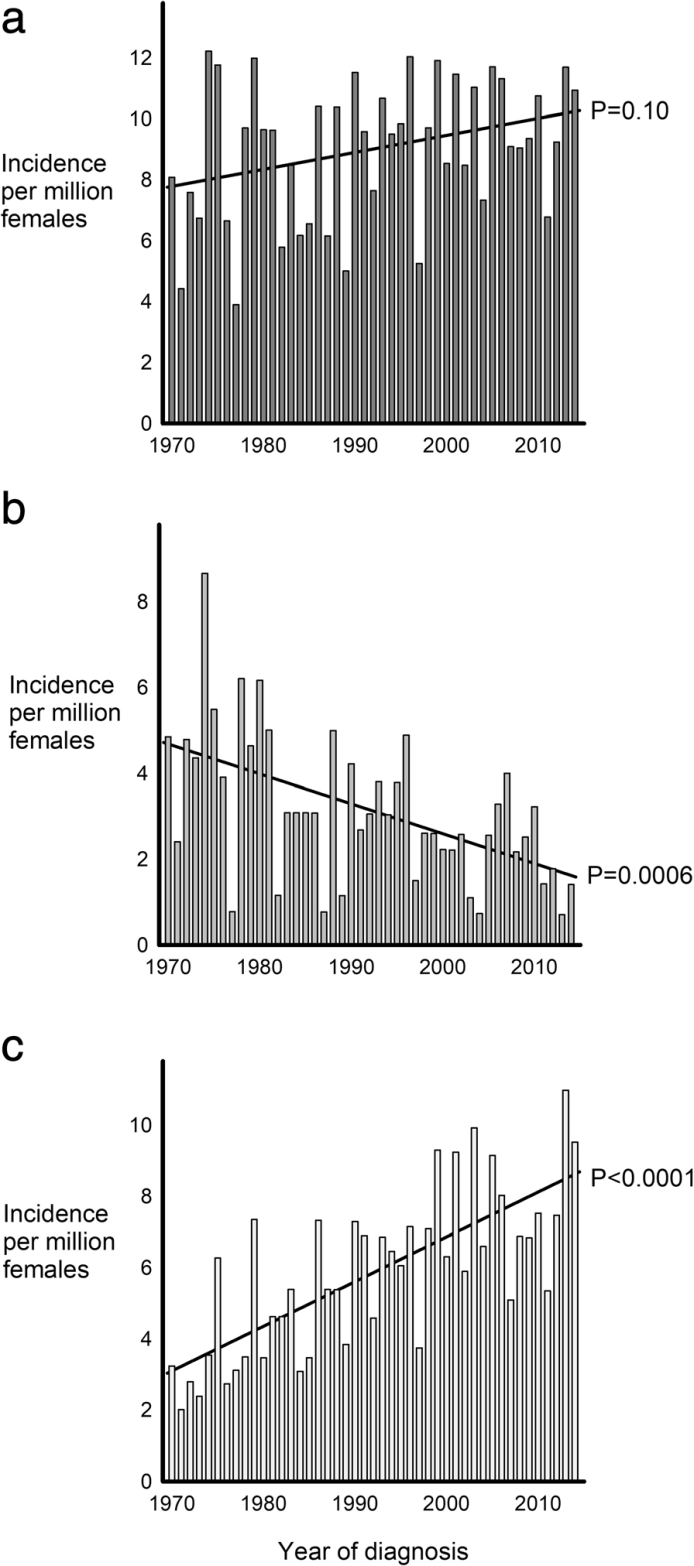
Incidence of Turner syndrome according to karyotype. Incidence of **a** all Turner syndrome (TS); **b** 45,X TS; and **c** TS with other karyotypes per million females during 1970–2014. Solid lines illustrate time trend in incidence during the 1970–2014. *P*-values indicate the significance level of time trend in incidence

During the study period an average of 2,648,000 females in Denmark were at risk of being diagnosed with a SCA. The average annual number of diagnosed TS was 24. Thus, the average incidence of TS was 9.0 per million females. The average number of 45,X TS and TS with other karyotypes than 45,X (“other” TS) diagnosed annually was 8 and 16, respectively, leading to an average incidence of 3.1 45,X TS and 5.9 “other” TS per million females. The incidence among all TS was stable during the study period (*P* = 0.10), whereas the incidence was decreasing for 45,X TS (*P* = 0.0006) and increasing for “other” TS (*P* < 0.0001) (Fig. 2).

**Fig. 3:**
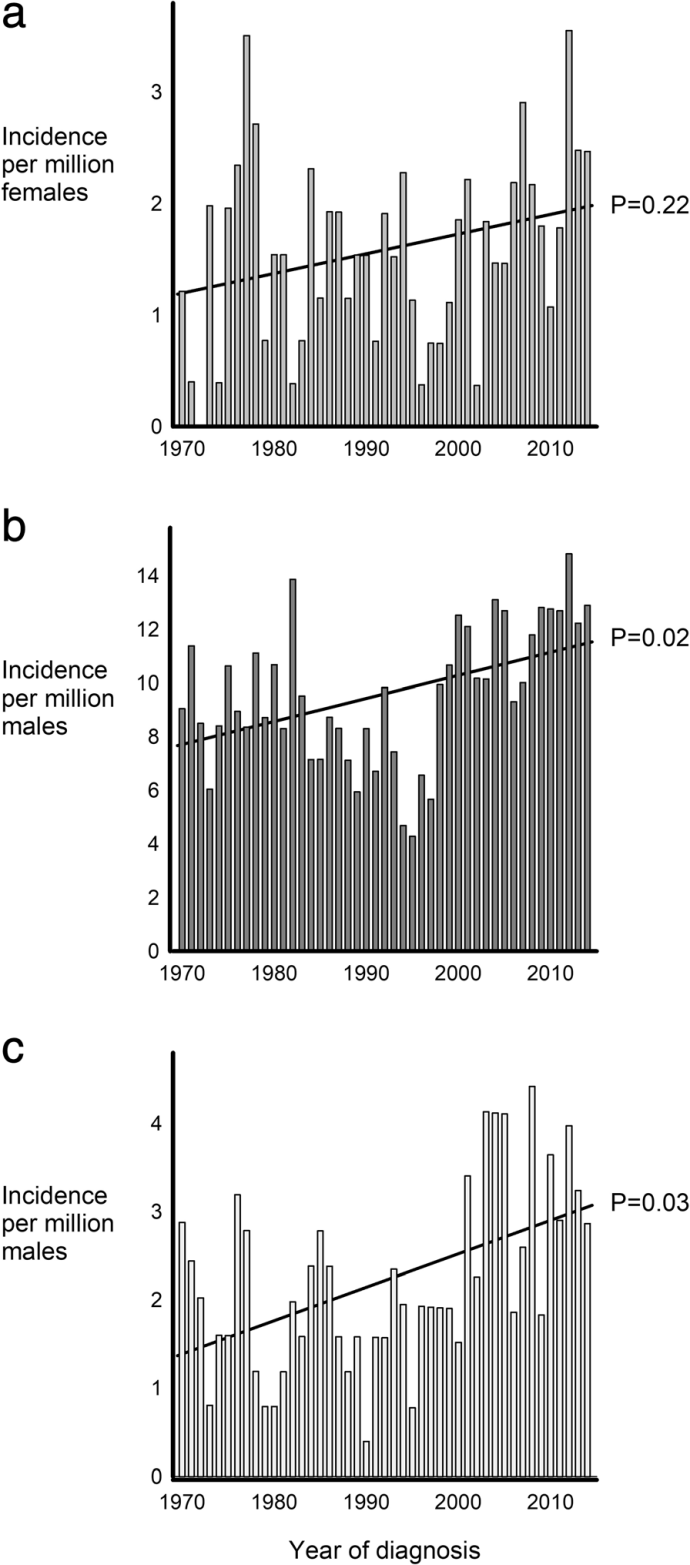
Incidence of Triple X syndrome, Klinefelter syndrome and Double Y syndrome. Incidence of **a** Triple X syndrome; **b** Klinefelter syndrome; and **c** Double Y syndrome per million females or males during 1970–2014. Solid lines illustrate time trend in incidence during the 1970–2014. *P*-values indicate the significance level of time trend in incidence

An average of four Triple X was diagnosed each year during the study period leading to an incidence of 1.6 Triple X per million females. No change in incidence among Triple X was observed during the study period (*P* = 0.22) (Fig. 3a).

The average number of males in Denmark being at risk of being diagnosed with a SCA each year during 1970–2014 was 2,589,000, and annually an average of 24 KS and 6 Double Y were diagnosed. Thus, the average incidence of KS and Double Y were 9.4 and 2.2 per million males during the study period. The incidence was increasing for KS (*P* = 0.02) as well as for Double Y (*P* = 0.03) during the study period (Fig. 3b and c).

### Prevalence among newborns

**Fig. 4:**
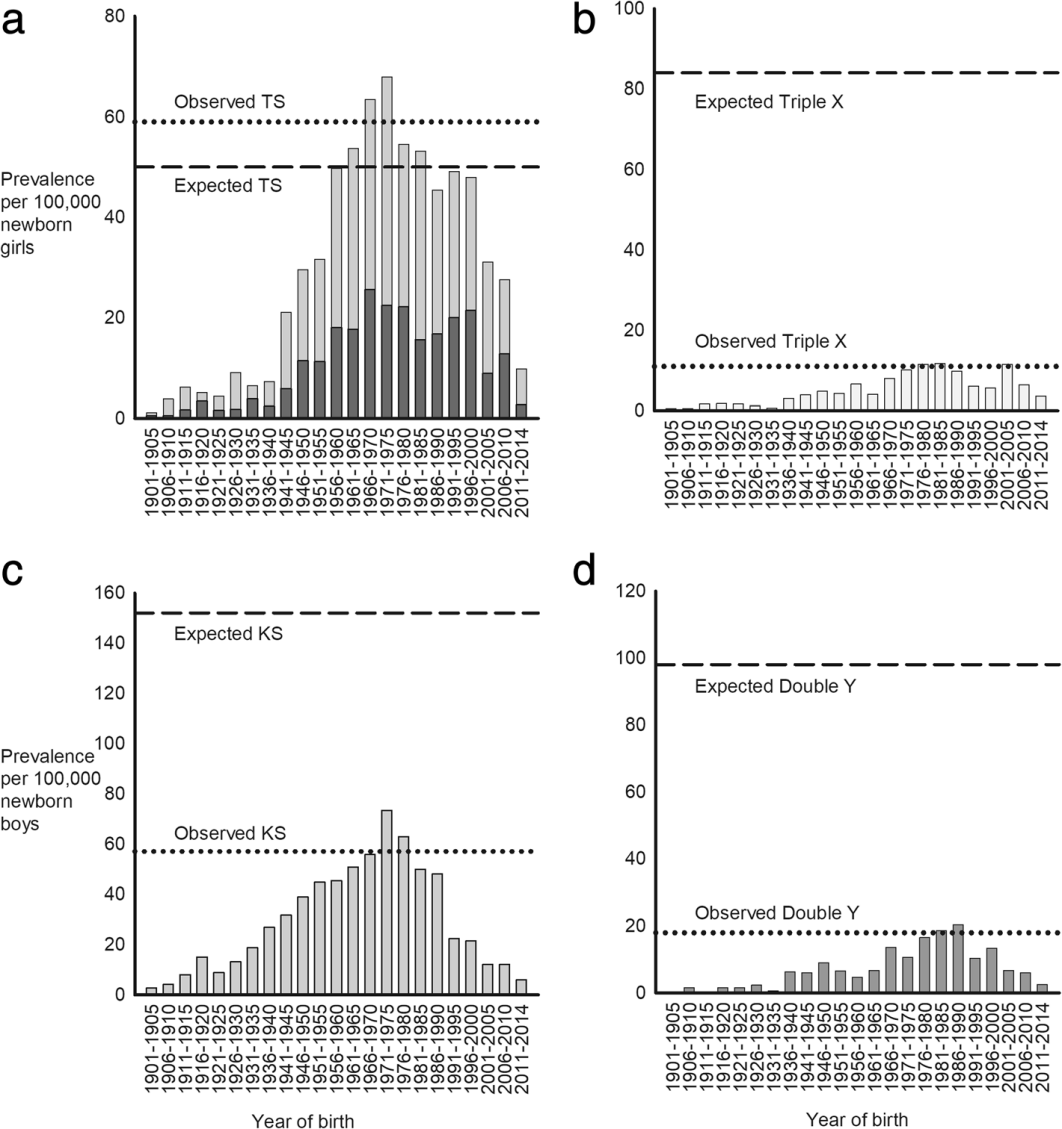
Prevalence of sex chromosome abnormalities among newborns. **a** Prevalence of Turner syndrome (TS). Black bars indicates the prevalence of 45,X TS among all TS; **b** Triple X syndrome (Triple X); **c** Klinefelter syndrome (KS); and **d** Double Y syndrome (Double Y) during 1901–2014. Dashed lines indicate the expected prevalence and dotted lines indicate the observed maximum average prevalence of TS, Triple X, KS and Double Y per 100,000 newborn females or males

Median year of birth among the SCAs was as follows: 1) TS: 1971 (range: 1885–2014); 2) Triple X: 1975 (range: 1895–2014); 3) KS: 1965 (range: 1882–2013); and 4) Double Y: 1977 (range: 1899–2013). During 1901 to 2014, prevalence was divided into sub-periods according to differences in average prevalence. During 1961–1985 the maximum average prevalence of all TS was 59 per 100,000 newborn females (45,X TS: 21 per 100,000 newborn females and “other” TS: 38 per 100,000 newborn females), thus higher than expected. During 1971–1990 the maximum average prevalence of Triple X was 11 per 100,000 newborn females, corresponding to 13% of the expected prevalence. The maximum average prevalence of KS was observed during 1961–1990 being 57 per 100,000 newborn males, corresponding to 38% of the expected prevalence. Double Y had a maximum average prevalence of 18 per 100,000 newborn males during 1976–1990, corresponding to 18% of the expected prevalence (Fig. 4).

### Age at diagnosis

**Fig. 5:**
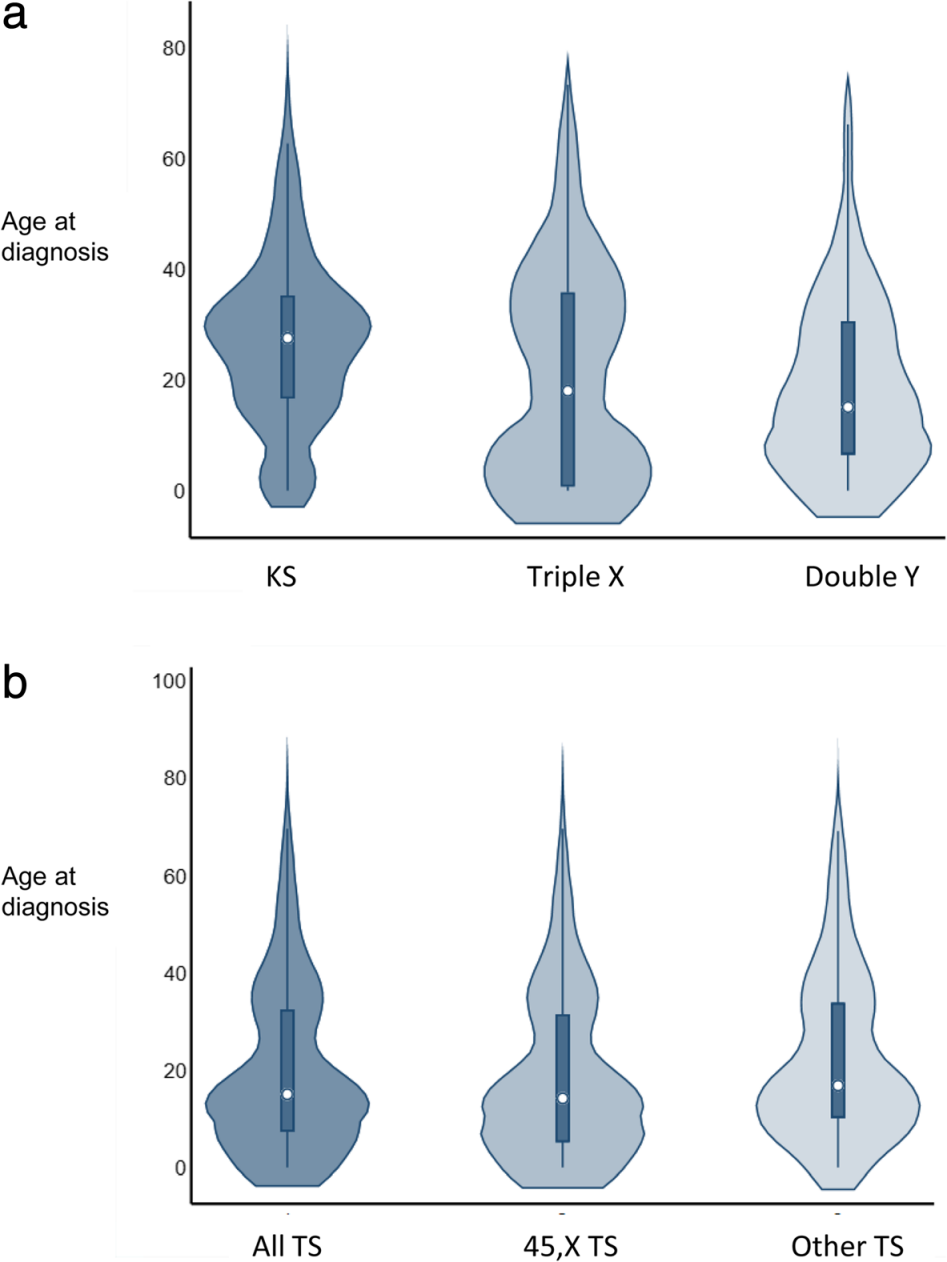
Age at diagnosis among sex chromosome abnormalities. Violin plots of age at diagnosis among **a** Klinefelter syndrome (KS), Triple X syndrome (Triple X) and Double Y syndrome (Double Y) and among **b** all Turner syndrome (TS), TS with a 45,X karyotype and TS with other karyotypes, diagnosed during 1970–2014. The small circle in the middle of the plot is median age, the dark rectangle depicts interquartile range, the thin dark lines depicts 95% confidence interval, and the density plot width equals frequency of age at diagnosis

The median age at diagnosis was for TS 15.1 (range: 0.0–85.4) years, for Triple X 17.9 (0.0–73.2) years, for KS 27.5 (0–82.8) years, and for Double Y 15.1 (0–70.7) years (Fig. 5a and b). 45,X TS was diagnosed with significantly less delay than TS with other karyotypes (median age: 45,X TS = 11.4 years versus “other” TS = 19.0 years) (*P* < 0.0001) (Fig. 5b). There was no change in age at diagnosis during 1970–2014 among all TS (*P* = 0.17), whereas age at diagnosis was decreasing among 45,X TS (*P* = 0.005). Age at diagnosis among TS with other karyotypes was stable (*P* = 0.99) during the study period, as well as among the other SCAs (Triple X: *P* = 0.28; KS: *P* = 0.72; and Double Y: *P* = 0.33). It is clear from the violin plots that the pattern of age at diagnosis is very different among different groups of SCAs (Fig. 5), with most KS being diagnosed much later than most TS, and with Triple X and Double Y being intermediate.

## Discussion

This population based study shows that the previously estimated prevalence of 50 TS per 100,000 may be an underestimate as we here present data showing a prevalence of 59 TS per 100,000 newborn females, corresponding to 1 per 1700. The karyotypic composition of the TS population is seemingly changing as the incidence of 45,X TS is decreasing and the incidence of TS with other karyotypes is increasing. Among KS and Double Y incidence is increasing as well, whereas it is stable among Triple X. Non-diagnosis remains extensive among KS, Triple X and Double Y. All TS eventually become diagnosed, although with a considerable delay.

The prevalence of the four forms of SCAs varied with a similar pattern during the study period. The marked increase in prevalence observed during the 1960’ties to the 1990’ties, possibly reflects that affected individuals from these birth cohorts were either born or reached puberty or fertil age at a time when karyotyping had become an established diagnostic procedure. However, comparing the maxiumum average prevalence with the expected prevalence of SCAs, the majority of KS (62%), Triple X (87%) and Double Y (82%) remain undiagnosed. In 2003, we reported that approximately 75% of expected KS was undiagnosed [2]. Although more with KS are diagnosed presently, we consider this far from satisfactory. In contrast, the average prevalence of TS exceeded the expected prevalence.

The population-based prevalence was steadily increasing for all SCAs during the study period caused by the build-up of the DCCR with continuing recruitment exceeding the rate of exit, a phenomenon known from other rare conditions as well [32]. The population-based prevalence will be stable when recruitment equals exit (death or emigration). Owing to increased mortality as well as increased rates of induced abortions among SCAs [33, 34] it will though remain lower than expected even with complete diagnosis of SCAs. However, it is difficult to ascertain how much prevalence is affected hereby. Only approximately 5% of pregnant women in Denmark have a prenatal karyotype performed [35], but they are of course selected as high-risk patients based on triple screening and non-invasive prenatal testing.

The incidence among all TS was stable during the study period. Interestingly, the incidence was steeply decreasing for 45,X TS and increasing for TS with other karyotypes. If this development continues it seems likely that 45,X TS are becoming extinct. Between 2004 and 2006, Denmark instituted a free prenatal screening program for Down syndrome (DS), in which over 90% of pregnant women in Denmark participate, and previously we have reported that approximately 40% of expected TS fetuses are detected by the DS screening [34]. The high induced abortion rate among prenatally detected 45,X TS fetuses likely contribute to the decreasing incidence of 45,X TS. Induced abortion among TS fetuses with other karyotypes are less pronounced [34], and a generally more favorable phenotype among these TS [36, 37] may prevent early diagnosis. A likely explanation for the increase in incidence among TS with other karyotypes may be that fertility treatment has become more common and thus more are diagnosed owing to fertility problems.

Late diagnosis as well as non-diagnosis of SCAs have been a continuous concern [25–27, 38] since SCAs are associated with increased morbidity and mortality [3, 5, 20, 23, 39], learning and/or behavioral disabilities [40–45] as well as reduced socioeconomic outcomes. Although there is lack of evidence regarding the influence of age at diagnosis on long-term outcomes, we believe that early diagnosis will provide better overall long-term outcome in SCAs by providing an opportunity for timely intervention against associated health problems as well as against learning and behavioral problems. Regrettably, the present study shows that delay in diagnosis remains a major problem (Fig. 5). Increased vigilance among health care professionals as well as a more liberal access to diagnostic tools was however hypothesized as having contributed to a decreasing diagnostic delay. As previously suggested for both TS and KS, neonatal screening programs would allow complete diagnosis of SCAs without diagnostic delay. Population-based, neonatal screening can be considered for conditions which have: 1) the magnitude to be an important health problem with a latent and early asymptomatic stage; and 2) has a well-understood natural history for which there are accepted treatments with associated facilities for diagnoses and treatment [46]. We consider these requirements fulfilled for SCAs. Due to the rarity of the syndromes it will, however, take a long time to demonstrate associations between early diagnosis, continuous specialized care and improved long-term outcomes.

The broad clinical spectrum observed among SCAs, ranging from overt to minimal or no apparent clinical features and only subtle symptoms [17, 47, 48] likely relates to the non-diagnosis and the delayed diagnosis. The fate of individuals with SCAs escaping diagnosis remains an enigma. Possibly, undiagnosed individuals experience a spectrum of similar challenges as those diagnosed, yet they might remain undiagnosed due to reluctance of seeking medical advice or due to lack of attention from health professionals. Based on our clinical experience, we believe that individuals diagnosed late in life more or less struggle with similar problems as those being diagnosed early in life. Recent data from UK among TS and Triple X support this hypothesis [36]. Here a large number of presumably undiagnosed 45,X and 47,XXX females were detected approximately about 250,000 examined females, presenting with typical features for both of these syndromes.

## Conclusions

The karyotypic composition of TS is changing with less 45,X and more mosaic TS being diagnosed, possibly due to increased frequency of legal abortions among 45,X TS. TS have a higher prevalence than previously anticipated as observed in 1 per 1700 newborn females. The majority of KS, Triple X and Double Y remain undiagnosed despite an increase in diagnostic activity among KS and Double Y. Delayed diagnosis is a continuous problem among all SCAs, and no change over time has been observed. We consider a newborn screening program as the only opportunity to reduce both diagnostic delay as well as non-diagnosis and with a potential to improve health and socioeconomics among individuals affected by SCAs.

## Abbreviations

CPR number: Civil personal registration number
DCCR: Danish Cytogenetic Central Registry
Double Y: Double Y syndrome
KS: Klinefelter syndrome
SCA: Sex chromosome abnormalilty
Triple X: Triple X syndrome
TS: Turner syndrome
